# Long-term associations between objective sleep quality and quantity and verbal memory performance in normal cognition and mild cognitive impairment

**DOI:** 10.3389/fnins.2023.1265016

**Published:** 2023-10-19

**Authors:** Eleni Skourti, Panagiotis Simos, Alexandros Zampetakis, Eirini Koutentaki, Ioannis Zaganas, Christina Alexopoulou, Alexandros Vgontzas, Maria Basta

**Affiliations:** ^1^Division of Psychiatry and Behavioral Sciences, School of Medicine, University of Crete, Heraklion, Greece; ^2^Computational Biomedicine Lab, Institute of Computer Science, Foundation for Research and Technology-Hellas, Heraklion, Greece; ^3^Department of Psychiatry, University Hospital of Heraklion, Crete, Greece; ^4^Division of Neurology and Sensory Organs, School of Medicine, University of Crete, Heraklion, Greece; ^5^Department of Intensive Care Unit, University Hospital of Heraklion, Crete, Greece; ^6^Sleep Research and Treatment Center, Department of Psychiatry and Behavioral Health, Penn State Health Milton S. Hershey Medical Center, College of Medicine, Pennsylvania State University, Hershey, PA, United States; ^7^Day Care Center for Alzheimer’s Disease “Nefeli”, University Hospital of Heraklion, Crete, Greece

**Keywords:** sleep fragmentation, time in bed, total sleep time, verbal memory, actigraphy, community-dwellers, mild cognitive impairment, longitudinal study

## Abstract

**Introduction:**

Although the link between sleep and memory function is well established, associations between sleep macrostructure and memory function in normal cognition and Mild Cognitive Impairment remain unclear. We aimed to investigate the longitudinal associations of baseline objectively assessed sleep quality and duration, as well as time in bed, with verbal memory capacity over a 7–9 year period. Participants are a well-characterized subsample of 148 persons (mean age at baseline: 72.8 ± 6.7 years) from the Cretan Aging Cohort. Based on comprehensive neuropsychiatric and neuropsychological evaluation at baseline, participants were diagnosed with Mild Cognitive Impairment (MCI; *n* = 79) or found to be cognitively unimpaired (CNI; *n* = 69). Sleep quality/quantity was estimated from a 3-day consecutive actigraphy recording, whereas verbal memory capacity was examined using the Rey Auditory Verbal Learning Test (RAVLT) and the Greek Passage Memory Test at baseline and follow-up. Panel models were applied to the data using AMOS including several sociodemographic and clinical covariates.

**Results:**

Sleep efficiency at baseline *directly* predicted subsequent memory performance in the total group (immediate passage recall: β = 0.266, *p* = 0.001; immediate word list recall: β = 0.172, *p* = 0.01; delayed passage retrieval: β = 0.214, *p* = 0.002) with the effects in Passage Memory reaching significance in both clinical groups. Wake after sleep onset time *directly* predicted follow-up immediate passage recall in the total sample (β = −0.211, *p* = 0.001) and in the MCI group (β = −0.235, *p* = 0.02). In the total sample, longer 24-h sleep duration was associated with reduced memory performance *indirectly* through increased sleep duration at follow-up (immediate passage recall: β = −0.045, *p* = 0.01; passage retention index: β = −0.051, *p* = 0.01; RAVLT-delayed recall: β = −0.048, *p* = 0.009; RAVLT-retention index:β = −0.066, *p* = 0.004). Similar indirect effects were found for baseline 24-h time in bed. Indirect effects of sleep duration/time in bed were found predominantly in the MCI group.

**Discussion:**

Findings corroborate and expand previous work suggesting that poor sleep quality and long sleep duration predict worse memory function in elderly. Timely interventions to improve sleep could help prevent or delay age-related memory decline among non-demented elderly.

## 1. Introduction

Sleep occupies about one third of a person’s lifetime. Many theories have been proposed regarding sleep function, most focusing on homeostatic processes crucial for physical, emotional and cognitive well-being (Zielinski et al., 2016). The link between sleep and memory function has been well validated in both animal models and human behavioral paradigms. NREM sleep microstructure (Slow Wave Sleep activity; SWS, spindles and K-complexes) is involved in Sleep-Dependent memory consolidation (SDC), a remodeling of brain activity between hippocampal circuits, prefrontal areas, amygdala and ventral tegmental area that promotes long-term information storage (Pace-Schott and Spencer, 2014). At the synaptic level, selective activation of dopaminergic and cholinergic neurons in the aforementioned brain areas during wakefulness (initial information encoding) and sleep may play a vital role in SDC, whereas sleep cycles facilitate synthesis of proteins involved in Long-Term Potentiation (Klinzing et al., 2019). Although the neurophysiological processes underpinning stabilization of memory traces is extensively studied, the specific associations between sleep and late-life cognition remain inconclusive, especially taking under consideration the significant changes documented in both domains among elderly.

Sleep architecture undergoes significant changes with advancing age. Age-related sleep changes involve reductions in sleep duration and sleep efficiency (SE) along with an overall increase in average time spent awake after sleep onset (WASO), particularly among old-old individuals (Junxin et al., 2017). Neural processes related to SDC attenuate: SWS activity, amplitude and total count of sleep spindles and K-complexes are significantly reduced. SDC disruption has been reported in older adults (Hornung et al., 2007), potentially related to increased sleep fragmentation which could, in turn, contribute to memory deterioration. Α recent meta-analysis focusing on age-related changes in the association between sleep and memory function indicated weakened effects of sleep on memory consolidation in older persons; sleep efficiency was a primary factor explaining the effect of SDC in this age group (Gui et al., 2017). Baseline self-reported long (≥10 h) and short sleep duration (<4 h) is associated with impaired follow-up global cognition in middle-aged and older persons (Ma et al., 2020) as well as with specific memory, language (fluency) and executive deficits (Dzierzewski et al., 2018). Poor sleep quality and increased rest-wake cycle variability estimated through actigraphy were associated with executive and memory impairment among old community-dwellers (Blackwell et al., 2006; Oosterman et al., 2009), whereas objective sleep discontinuity and subjectively assessed short sleep duration increased odds of dementia diagnosis and predicted cognitive deterioration in the long-term (Keage et al., 2012; Lim et al., 2013).

Apart from the age-associated sleep pattern alterations, sleep dysregulation is highly prevalent in patients diagnosed with Mild Cognitive Impairment (MCI) and dementia and represents a marker of disease severity and a putative risk factor for cognitive impairment (Pak et al., 2020). It is well established that sleep–wake disruption is further exacerbated in patients with MCI and Alzheimer’s Disease (AD), leading to additional deterioration in qualitative and quantitative sleep indices as verified by polysomnography and actigraphy studies. Persons diagnosed with MCI may show increased WASO, lower SE and average 24-h sleep duration along with reduced REM sleep and NREM sleep, compared to cognitively non-impaired individuals (Cai et al., 2020), although some studies failed to show statistically significant differences between cognitively normal and MCI persons on objectively assessed sleep duration indices (Basta et al., 2019). Sleep abnormalities in dementia patients entail further exacerbation of sleep continuity indices, increased incidence of insomnia-type symptoms, as well as excessive daytime sleepiness, although the exact pattern of sleep abnormalities depends on the type of dementia diagnosis (Cipriani et al., 2014). Persons diagnosed with MCI with prevailing memory deficits (amnestic subtype) were more likely to present low SE and increased WASO as well as decreased SWS activity amplitude and total spindles count. Also, actigraphy-based long sleep duration was associated with worse executive performance among MCI and dementia individuals in a large community-dwelling subsample of the Cretan Aging Cohort study (CAC; Basta et al., 2019), whereas other studies fail to report significant associations between specific cognitive domains and objective sleep quality/quantity (Scarlett et al., 2021).

The present longitudinal study addresses the scarcity of cohort studies investigating putative associations between sleep characteristics and memory in elderly without dementia (MCI or CNI), in view of the significant developmental changes in sleep micro-and macro structure and the well-documented relationship between sleep and cognition. Accordingly, the main objective of the current study was to assess both direct and indirect, long-term effects of objective, actigraphy-based sleep measures on memory performance assessed 7–9 years later. Path models allowed us to examine (a) direct effects of baseline objective sleep characteristics on subsequent memory performance by controlling for the effects of both baseline memory and subsequent objective sleep characteristics, and (b) indirect effects of baseline objective sleep characteristics on subsequent memory performance as mediated by individual objective sleep characteristics assessed at this later time point (controlling for the effects of baseline memory status). We hypothesized that (i) higher 24-h Total Sleep Time (TST) and Total Time in Bed (TiB) and higher WASO at baseline would predict lower memory capacity at follow-up, (ii) higher baseline SE would predict higher memory capacity at follow-up, (iii) the long-term effects of sleep on memory may occur both directly and indirectly as they evolve over time, and (iv) these effects would be evident in the total sample but predominantly among persons who were diagnosed with MCI at baseline.

## 2. Materials and methods

A subsample of 148 participants from the CAC, a large cohort study conducted in the island of Crete, Greece was retested within 7–9 years (mean interval: 7.12 years, SD = 0.92) from the initial assessment. All participants underwent extensive neuropsychological, neuropsychiatric, and neurological examination, as well as 3-day actigraphy recording at baseline (Phase I & II) and follow-up (Phase III). Phase I & II procedures were approved by the Bioethics Committee of the University Hospital of Heraklion, Crete (Protocol Number: 13541, 20-11-2010), whereas current study protocol was approved by the Ethics Committee of the University of Crete (number of approval: 61/9-3-2020). For a detailed description of Phases I, II & III see Zaganas et al. (2019) and Basta et al. (2023).

*Phase I & II (Baseline)*. Consenting participants (*n* = 3.200, ≥60 years old) were recruited from selected Health Care Primary settings in the district of Heraklion. Additional eligibility criteria were absence of terminal illnesses and/or movement impairment. Sociodemographic information, anthropometric measurements and medical history was collected by trained physicians and the Greek version of the Mini Mental State Examination was administered (with a cut-off score of 23/24 points; Fountoulakis et al., 2000). The final cohort, after excluding participants with crucial missing data comprised 3.140 individuals (mean age at baseline: 73.7 ± 7.8 years; Zaganas et al., 2019).Out of the 636 participants with MMSE score < 24 points, 344 consented to participate in a comprehensive neuropsychological examination, while a control group (161 out of 181 individuals with, MMSE score ≥ 24 points) matching the low MMSE group in terms of sociodemographic characteristics was formed based on a proportional stratification procedure (for more information see Basta et al., 2019, 2023). Participants were then classified into three diagnostic categories: CNI (*n* = 146), MCI (*n* = 231) and Dementia (*n* = 128) according to the International Working Group criteria for MCI diagnosis (Winblad et al., 2004) and the Diagnostic Statistical Manual for Mental Disorders (4^th^ edition; DSM-IV) for dementia diagnosis.

*Phase III (Follow-up)*. All participants who met formal criteria for MCI (*n* = 231) and CNI individuals (*n* = 146) at baseline were invited to participate in Phase III (follow-up). Follow-up assessments took place between October 2020 and August 2022. Participants came from 11 districts in the prefecture of Heraklion and contacted by telephone. In total, 151 individuals completed the evaluation and provided useful data, achieving an acceptable response rate (55.1%). Attrition rate (inability to participate for any reason) was estimated to 58.9%. Compared to the total participant pool (all persons in the CNI and MCI groups on Phase II, *n* = 377), those who were followed-up were younger (72.8 vs. 77.2 years, *p* < 0.001), more likely to be women (77.5% vs. 63.3%, *p* = 0.004) and less likely to live alone (*p* = 0.03). Followed-up persons have achieved more years of education, although the difference was not found statistically significant (*p* = 0.059), whereas the two groups were comparable in terms of geographic origin (*p* = 0.4), previous occupation (*p* = 0.1) and physical illnesses (*p* = 0.9). Neuropsychological and medical examination was conducted at participants’ homes. Procedures implemented during baseline and follow-up were the same rendering results comparable between the two phases (for a detailed description of the study protocol, see also Basta et al., 2023).

### 2.1. Sociodemographic and medical information

Demographic information (age, gender, educational and family status) was recorded in the context of a semi-structured interview. Anthropometric measurements (BMI), critical physical comorbidities (hypertension, diabetes, heart, pulmonary, hematological or liver diseases, gastrointestinal conditions, hyper/hypothyroidism, cancer, arthritis), presence of sleep apnea, use of SSRIs, benzodiazepines and other anxiolytics were also recorded at both measurement points. A binary categorical variable was constructed based on the number of physical morbidities reported by each participant (coded with 0 for ≤4 and 1 for ≥5 physical illnesses).

### 2.2. Insomnia-type symptoms and sleep apnea

Penn State Sleep Questionnaire (PSSQ-12) was administered in order to assess the presence of subjective sleep disturbances and sleep apnea (Basta et al., 2018). Insomnia-type symptoms included difficulty initiating sleep, difficulty maintaining sleep and early morning awakening. Answers to three single questions requiring a yes/no response (“Do you have difficulty falling asleep?”; “Do you have difficulty staying asleep?”; “Do you wake up in the morning earlier than desired?”) were used to construct three separate categorical variables pertaining to each insomnia symptom. A new binary variable indicating severity of self-reported insomnia symptoms was finally constructed (0 = at least one insomnia symptom and 1 = two or more insomnia symptoms).Symptoms of sleep apnea were estimated using two questions (requiring a yes/no response): “Have you ever noticed/Have you been told that you stop breathing/breathe irregularly while asleep?” and/or “Do you know/Have you been told that you snore during sleep to a moderate/severe degree?.” Presence of sleep apnea (indicated by a binary variable) was determined on the basis of at least one positive response to the aforementioned questions.

### 2.3. Objectively assessed sleep indices (baseline and follow-up)

Sleep variables were estimated based on a consecutive 3-day actigraphy recording (baseline and follow-up; Basta et al., 2019, 2023). Participants were instructed to wear an actigraph (Actilife v6.9.5, GT3XP model, Pensacola, FL, United States) on their non-dominant hand, while simultaneously keeping sleep diaries and reporting “time in bed” and “time out of bed” and napping periods (>20 min) throughout the day, on a daily basis. Periods of movement absence that were not recorded in sleep diaries as sleep periods were removed from further analysis. Analysis of actigraphy data was performed using ActiLife 6 software (Actigraph LLC) and the following quality/quantity sleep variables were estimated: night sleep efficiency (SE), night wake after sleep onset time (WASO), night and 24-h total sleep time (TST) and finally, night and 24-h time spent in bed (TiB). Recording procedures were similar in both phases and took place during weekdays. Recording period started at 11.00 on the day that the actigraph was attached to the participant’s hand and ended 24 h later. Participants with fewer than 3 days of actigraphy recording and those with relatively low night sleep periods (average night TST < 180 min) were excluded from further analysis.

### 2.4. Neuropsychological assessment (baseline and follow-up)

Verbal memory was assessed at baseline and follow-up using two complementary tests: (1) the Greek adaptation of the Rey Auditory Verbal Learning Test (RAVLT) consisting of a 15-item list assessing immediate and delayed recall, as well as retention ability, defined as the number of correctly recalled words on immediate recall (5^th^ trial) vs. the number of words recalled after a 30-min delay period (7^th^ trial; Constantinidou et al., 2014) and (2) the Passage Memory test from the Greek Memory scale assessing immediate and delayed recall for two short stories (Simos et al., 2011). Raw scores were transformed into age and education-adjusted standard (z) scores using the regression method proposed by Ivnik et al. (1992), based on Greek normative data reported elsewhere (Constantinidou et al., 2014). Briefly, the standardization sample comprised 550 adults (321 women) aged 17–86 years (mean = 46.8, SD = 16.7 years) from the general population without history of neurological or psychiatric disorder (including learning disability) who did not report significant cognitive difficulties. Residence was urban (65.3%), small town (16.0%), and rural (18.7%) and had achieved an average of 12.33 years of education (SD = 3.91, range 1–24). Standard scores were computed independently for performance at each measurement point (adjusting for the increased age at follow up).Significant positive correlations were found between the aforementioned memory indices and the MMSE score at both measurement points (baseline: Pearson’s r > 0.3, *p* < 0.001 and follow-up: Pearson’s r: 0.4–0.76, p < 0.001), as well as longitudinally (baseline MMSE performance and all follow-up memory scores: Pearson’s r > 0.5 for immediate and delayed Passage Memory scores; *p* < 0.001), indicating the presence of significant congruence between measures of memory and global cognition.

### 2.5. Neuropsychiatric evaluation (baseline and follow-up)

Depression symptoms were evaluated through a semi-structured clinical interview conducted by specialized physicians and neuropsychologists. Self-reported questionnaires assessing for depression symptoms (15-item Geriatric Depression scale and the Center for Epidemiologic Studies Depression Scale) were administered and current psychotropic medication use was also recorded. Depression diagnosis on baseline or follow-up (indicated by a score of 1 on a binary variable) was established based on current use of antidepressants, clinical interview and scores on self-report questionnaires according to the DSM (4^th^Edition) and DSM (5^th^Edition) for Phase I & II and Phase III, respectively. Accordingly, anxiety diagnosis was based upon the current use of psychotropic substances (benzodiazepine, anxiolytics), subjective feelings of distress (evaluated with the anxiety subscale of the Hospital Anxiety Depression Scale;HADS-A) and clinical interview, following the DSM (4^th^ and 5^th^ Edition) for baseline and follow-up assessments. A categorical variable indicating the presence (=1) or absence (=0) of clinical anxiety manifestations was subsequently formed.

### 2.6. Statistical analysis

SPSS 29 (IBM SPSS, Version 29.0, Armonk, NY: IBM Corp) and Analysis of Moment Structures (AMOS) were used for statistical analysis. Continuous variables on each measurement point are presented as means and standard deviations, and categorical variables as frequencies and percentages. Change in continuous variables between baseline and follow-up was assessed using paired samples t-test and the Chi square test of independence for categorical variables. Pearson correlations between sleep and neuropsychological indices were also computed. Statistical significance level was set to *p* < 0.05.

**Figure 1:**
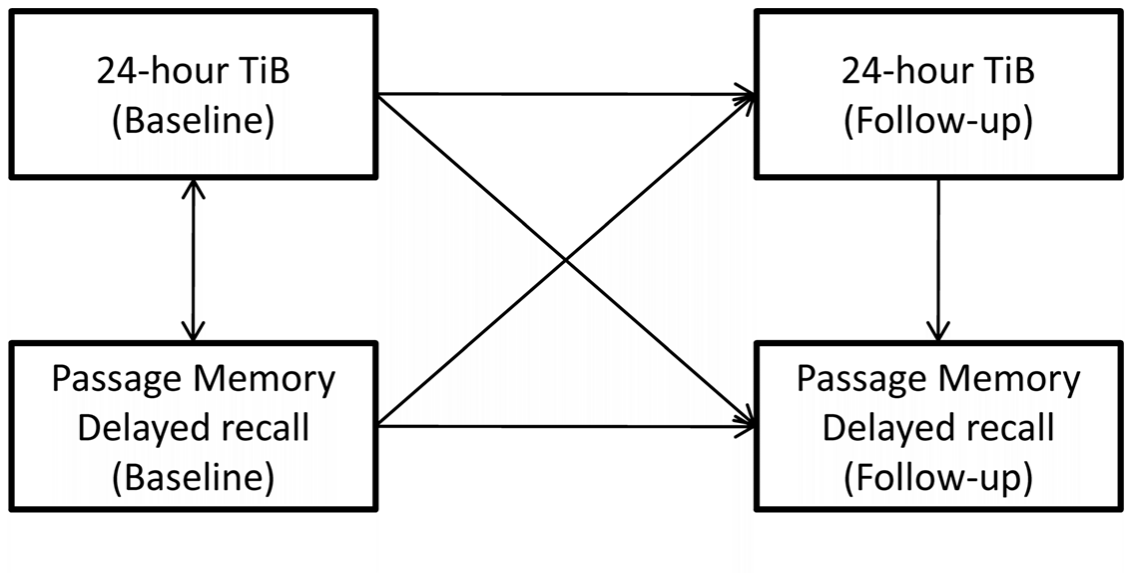
An example of the basic path model addressing the main objective of the study. In this example, age- and education-adjusted delayed passage recall at follow-up (outcome) is predicted by (i) the indirect effect of baseline 24-h time in bed (TiB) through follow-up 24-h TiB (main effect of interest), (ii) the indirect effect of baseline delayed passage recall through follow-up 24-h TiB, (iii) the direct effect of follow-up, 24-h TiB. Final models included several demographic and clinical covariates (not shown).

Significant direct and indirect relationships between baseline sleep indices and follow-up memory performance were examined through path models in AMOS. Basic models (i.e., without covariates) were initially computed including a single sleep and a single memory variable at baseline and the corresponding sleep and memory variables at follow-up (Figure 1). In the presence of either a significant direct effect of the baseline sleep index on follow-up memory index or a significant indirect effect of the baseline sleep index on the follow-up memory index through the corresponding follow-up sleep index, the following covariates were entered into the model: BMI, education (in years), age (in years), gender, depression and anxiety diagnosis, presence of ≥5 physical illnesses, the presence of at least one insomnia-type symptom self-reported sleep apnea and benzodiazepine use. All covariates were assessed at baseline.

Direct and indirect effects are expressed as standardized beta coefficients (β), unless otherwise stated. Statistical significance level was set to *p* < 0.05. Models with adequate fit to the data (indicated by Chi square test *p* ≥ 0.05; Goodness of Fit Index (GFI) >0.90; Root Mean Square Error of Approximation (RMSEA) ≤ 0.08) are presented in the main text, whereas models presenting slight deviations from the criteria mentioned above are presented in the Supplementary section.

To aid interpretation of the results of the path models, we performed two additional sets of analyses. Firstly, we compared the subgroup of CNI participants who met criteria for MCI or dementia at follow up (“progressors”) with those who remained cognitively non-impaired (“non-progressors”) on sleep indices at baseline and again at follow-up using t-tests. Similarly, we compared the subgroup of MCI participants who met criteria for dementia at follow up (“progressors”) with those who continued to meet MCI criteria(“non-progressors”). Secondly, we assessed the linear association between baseline sleep variables and change in raw memory scores over time using Pearson correlations.

## 3. Results

### 3.1. Sample sociodemographic and clinical characteristics

Complete longitudinal data were available on 148 persons (77.7% women with mean age at baseline of 72.8 years). The majority was residing in rural areas of Heraklion (85.1%) and had completed 6 or less years of formal schooling (91.9%). Depression diagnosis incidence and use of psychotropic medication (SSRIs, benzodiazepines and anxiolytics) at baseline was estimated at 31.1 and 29.7%, whereas benzodiazepine use was estimated to 10.1 and 11.5% at baseline and follow-up, respectively. The test–retest delay period ranged between 7–9 years. Sociodemographic and medical characteristics of the total sample are presented in Table 1.

**Table 1:** Sociodemographic and clinical characteristics of the total sample (*N* = 148) at each measurement point.

	Baseline	Follow-up
Age (mean, SD)	72.8 (6.7)	80.9 (6.6)*
Females (%, *n*)	77.7 (115)	
Rural origin (%, *n*)	85.1 (126)	
Education (years, mean, SD)	5.12 (2.9)	
≥5 Physical illnesses (%, *n*)	9.5 (14)	31.1 (46)
BMI (mean, SD)	30.5 (4.3)	30.6 (5.6)
Living alone (%, *n*)	20.3 (30)	27.7 (41)*
Depression Diagnosis (%, *n*)	31.1 (46)	36.5 (54)*
Anxiety Diagnosis (%, *n*)	30.4 (45)	30.4 (45)
Psychotropic use (%, *n*)	29.7 (44)	42.9 (63)*
Benzodiazepine use (%, *n*)	10.1 (15)	11.5 (17)*
Cognitive status (%, *n*)		
*CNI*	46.6 (69)	26.4 (39)*
*MCI*	53.4 (79)	45.9 (68)
*Dementia*	–	27.7 (41)
MCI type (%, *n*)		
*Single-domain*		
*Amnesic*	26.6 (21)	20.6 (14)
*Non amnesic*	16.5 (13)	27.9 (19)
*Multi-domain*	57.0 (45)	51.5 (35)

*Statistically significant change between baseline and follow-up (paired samples t-test for continuous variables and Chi square test for proportions; *p* < 0.05).

Overall, 69 and 79 individuals were diagnosed as CNI and MCI cases at baseline. CNI participants were significantly younger compared to their MCI counterparts (mean age at baseline: 70.4 ± 6.24 vs. 74.9 ± 6.33 years, *p* < 0.001) and had completed more years of formal education, although the difference was not statistically significant (5.6 vs. 4.7 years, *p* = 0.08). The two groups were comparable in terms of gender ratio (78.3% vs. 77.2%, *p* = 0.9), BMI (31.1 ± 4.23 vs. 30.0 ± 4.39, *p* = 0.1), living alone(21.7% vs. 19.0%, *p* = 0.7), presence of ≥5 physical illnesses (8.7% vs. 10.1%, *p* = 0.8), depression diagnosis (27.5% vs. 34.2%, *p* = 0.4), and SSRI/benzodiazepine/anxiolytic use at baseline (25.3% vs. 34.8%, *p* = 0.2, respectively). The frequency of anxiety diagnosis did not vary significantly over time in the MCI and CNI groups (*p* = 0.5 and p = 0.2, respectively).

### 3.2. Memory performance at baseline and follow-up

There was a significant decline on all memory indices in the total sample between baseline and follow-up, as shown in Table 2. In general, average baseline z-scores of the total sample on memory tasks were in the average to low-average range with the exception of the word list Memory Retention Index (RAVLT; z = −1.56) which was largely due to the MCI group performing, on average, over two standard deviations below population average.

**Table 2:** Memory performance of the total sample and stratified by clinical group at each measurement point (values are mean [SD] age– and education-adjusted z scores).

	Total sample (*n* = 148)		CNI (*n* = 69)		MCI (*n* = 79)	
	Baseline	Follow-up	Baseline	Follow-up	Baseline	Follow-up
PM: immediate recall	−0.68 (0.94)	−1.35 (0.95)*	−0.23 (0.92)	−0.89 (0.97)*	−1.08 (0.76)	−1.75 (0.73)*
PM: delayed recall	−0.71 (1.00)	−1.43 (1.03)*	−0.18 (0.98)	−0.84 (1.06)*	−1.16 (0.77)	−1.94 (0.65)*
PM: retention index	−0.37 (1.93)	−2.26 (2.32)*	0.28 (1.50)	−0.90 (2.19)*	−0.94 (2.09)	−3.44 (1.70)*
RAVLT:immediate recall	−0.69 (0.86)	−1.25 (1.27)*	−0.32 (0.83)	−0.58 (1.08)	−1.00 (0.76)	−1.84 (1.14)*
RAVLT: delayed recall	−0.57 (1.05)	−1.04 (1.09)*	−0.10 (0.93)	−0.49 (1.13)*	−0.99 (0.97)	−1.53 (0.77)*
RAVLT: retention index	−1.56 (1.74)	−2.80 (2.01)*	−0.91 (1.29)	−1.81 (1.89)*	−2.12 (1.89)	−3.67 (1.69)*

*Significant change between baseline and follow-up (paired sample t-tests; p < 0.001). PM, Passage Memory; RAVLT, Rey Auditory Verbal Learning Test.

On follow-up, average scores of the CNI group remained within 1 SD from the population mean, with the exception of both Memory Retention Indices, where they displayed an average decline of approximately two (RAVLT) and one SDs (Passage Memory). On all other indices standard scores declined on follow-up by ≤0.66 SDs (*p* < 0.001). The MCI group also showed significant reduction in all memory indices (p < 0.001) ranging between 0.54 (RAVLT- delayed recall) and 2.5 SDs (Passage Memory Retention Index). These results are in line with the observation that only 27 (39.1%) and 6 (8.7%) of CNI persons were diagnosed with MCI and dementia on follow-up, respectively, whereas 41(51.9%)persons in the MCI group continued to meet MCI criteria, and 35 (44.3%) developed dementia.

### 3.3. Sleep variables at baseline and follow-up

In the total sample, as shown in Table 3, sleep quality indices (SE and WASO) improved with advancing age. This trend was also evident in each of the two clinical groups. Regarding sleep quantity indices, there was a significant increase in 24-h TST in the total sample (*p* < 0.001), which was largely due to participants in the MCI group (average increase = 66.4, SD = 91.6 min; *p* < 0.001; CNI: mean = 0.4, SD = 86.6 min; *p* = 0.1). Whereas 24-h TiB remained stable in the total sample (*p* = 0.9), there were notable group differences in the direction of change over time: TiB increased significantly in the MCI group (mean = 31.5, SD = 102 min; *p* = 0.007) and decreased among CNI participants (mean: 37.8, SD = 104 min; *p* = 0.004). A modest increase in the report of insomnia-type symptoms in both groups did not reach significance (*p* = 0.7 and *p* = 0.09 in the CNI and MCI groups, respectively), whereas self-reported sleep apnea decreased in both groups between the two time points (*p* = 0.01 and p < 0.001 among MCI and CNI persons, respectively).Of the 41 persons with dementia, 37 met clinical criteria for probable Alzheimer’s Disease, one person was identified as having mixed dementia and three presented with LBD-type profile, according to the NINCDS-ADRDA (McKhann et al., 1984), the NINDS-AIREN (Roman et al., 1993) and the DLB Consortium criteria (McKeith et al., 2005), respectively.

**Table 3:** Objective and subjective sleep parameters of the total sample and stratified by clinical group at each measurement point.

	Total sample (*n* = 148)		CNI (*n* = 69)		MCI (*n* = 79)	
	Baseline	Follow- up	Baseline	Follow- up	Baseline	Follow- up
Sleep efficiency	81.0 (8.21)	86.0 (5.35)*	83.0 (6.87)	86.4 (4.53)*	79.6 (8.96)	85.2 (5.95)*
WASO	78.3 (38.18)	66.2 (27.47)*	75.7 (34.77)	59.3 (20.59)*	80.6 (41.0)	72.3 (31.18)
24-h TiB	553.4 (84.62)	552.6 (88.15)	559.3 (80.16)	521.5 (80.48)*	548.3 (88.52)	579.8 (85.97)*
24-h TST	440.4 (66.0)	476.0 (81.18)*	452.4 (66.26)	452.8 (73.42)	429.9 (63.61)	496.3 (82.61)*
≥2 Insomnia symptoms (%, n)	20.3 (30)	29.1 (43)	23.2 (16)	33.3 (23)	17.7 (14)	25.3 (20)
Sleep apnea (%,n)	16.8 (25)	19.0 (28)*	21.7 (15)	23.2 (16)*	12.6 (10)	15.2 (12)

*Significant change between baseline and follow-up (paired sample t-tests for continuous variables and Chi square tests for proportions; *p* < 0.05). Values are mean (SD) unless otherwise specified. WASO, Wake After Sleep Onset time; TiB, Total Time in Bed; TST, Total Sleep Time.

### 3.4. Bivariate associations between sleep indices and memory performance

Bivariate correlations between sleep indices at both measurement points and memory performance at follow-up are presented in Table 4. In general, sleep indices exhibited weak yet significant positive correlations between baseline and follow-up (*p* < 0.05) in the total sample. These correlations were notably stronger in the CNI group indicating greater individual stability of these sleep indices over time.

**Table 4:** Pearson correlations between objective sleep indices (baseline, follow-up) and memory performance (age and education-adjusted z-scores at follow-up) in the total sample, MCI and CNI groups.

	Sleep efficiency		WASO		24-h TiB		24-h TST	
Memory performance	Baseline	Follow-up	Baseline	Follow-up	Baseline	Follow-up	Baseline	Follow-up
Total sample	0.287***^1^		0.196***^1^		0.217**^1^		0.177*^1^	
RAVLT: Immediate recall	0.155	0.013	−0.141	−0.207*	−0.163*	−0.490***	−0.094	−0.447***
RAVLT: Delayed recall	0.176*	0.022	−0.161	−0.197*	−0.178*	−0.429***	−0.094	−0.377***
RAVLT: Retention index	0.138	0.056	−0.126	−0.258**	−0.148	−0.504***	−0.069	−0.435***
PM:Immediate recall	0.241**	−0.037	−0.217**	−0.138	−0.105	−0.329***	0.018	−0.300***
PM: Delayed recall	0.254**	0.052	−0.218*	−0.202*	−0.172*	−0.313***	−0.049	−0.258**
PM: Retention index	0.253**	0.114	−0.247**	−0.259***	−0.152	−0.389***	−0.017	−0.316***
MCI	.197^1^		.159^1^		0.322**^1^		0.236*^1^	
RAVLT: Immediate recall	0.043	−0.082	−0.135	−0.096	−0.202	−0.462***	−0.179	−0.432***
RAVLT: Delayed recall	0.084	−0.149	−0.117	−0.014	−0.113	−0.388**	−0.055	−0.350**
RAVLT: Retention index	0.049	−0.104	−0.118	−0.092	−0.152	−0.455***	−0.105	−0.420***
PM:Immediate recall	0.166	−0.079	−0.200	−0.046	−0.033	−0.198	0.092	−0.174
PM: Delayed recall	0.147	−0.017	−0.185	−0.085	−0.108	−0.118	−0.022	−0.075
PM: Retention index	0.161	0.017	−0.209	−0.133	−0.120	−0.299**	−0.013	−0.238*
CNI	0.419***^1^		0.248*^1^		.161^1^		.234^1^	
RAVLT: Immediate recall	0.094	0.006	−0.111	−0.131	−0.254*	−0.314**	−0.297**	−0.447***
RAVLT: Delayed recall	0.106	0.077	−0.187	−0.213	−0.359**	−0.333**	−0.279**	−0.377***
RAVLT: Retention index	0.052	0.153	−0.099	−0.309**	−0.260*	−0.384***	−0.304**	−0.435***
PM: Immediate recall	0.188	−0.126	−0.231	−0.030	−0.270*	−0.235*	−0.249*	−0.300***
PM: Delayed recall	0.223	−0.002	−0.265*	−0.117	−0.372**	−0.220	−0.202	−0.258**
PM: Retention index	0.197	0.125	−0.311**	−0.216	−0.335**	−0.237*	−0.188	−0.316***

TiB, Total Time in Bed; TST, Total Sleep Time; PM, Passage Memory; RAVLT: Rey Auditory Verbal Learning Test. *p ≤ 0.05, **p ≤ 0.01, ***p ≤ 0.001. ^1^Pearson’s correlation coefficients between baseline and follow-up for each sleep index.

Regarding associations between baseline sleep indices and follow-up memory performance in the total sample, only those involving sleep quality indices reached significance, primarily with Passage Memory scores (*p* < 0.01): higher SE and lower WASO predicted higher memory scores at follow-up. There were also weaker, albeit significant, negative correlations between baseline 24-h TiB and immediate and delayed recall measures (both memory tasks; *p* < 0.05). In the CNI group, significant negative correlations were noted between WASO, 24-h TiB, and 24-h TST and several memory indices. In the MCI group, these associations did not approach significance.

Associations between sleep and memory indices at follow-up were more robust, entailing negative correlations between WASO, 24-h TiB and 24-h TST with nearly all memory indices. In both clinical groups only correlations involving 24-h TiB and 24-h TST reached significance, although a significant negative correlation between WASO and RAVLT Retention Index was found among CNI participants (*p* = 0.01).

### 3.5. Direct and indirect paths between baseline sleep indices and follow-up memory performance in the total sample

Α total of 24 path models were conducted. All model fit criteria were met by 10 models, whereas significant direct or indirect effects of baseline sleep on follow-up memory scores were found in 6 additional models demonstrating marginally acceptable fit (*p* < 0.05 for all chi-square indices). These effects are described in detail separately for sleep quality (SE, WASO) and sleep duration indices (24-h TiB, 24-h TST) in sections 3.5.1 and 3.5.2.

#### 3.5.1. Baseline sleep quality indices directly predict long-term verbal episodic memory performance

**Figure 2:**
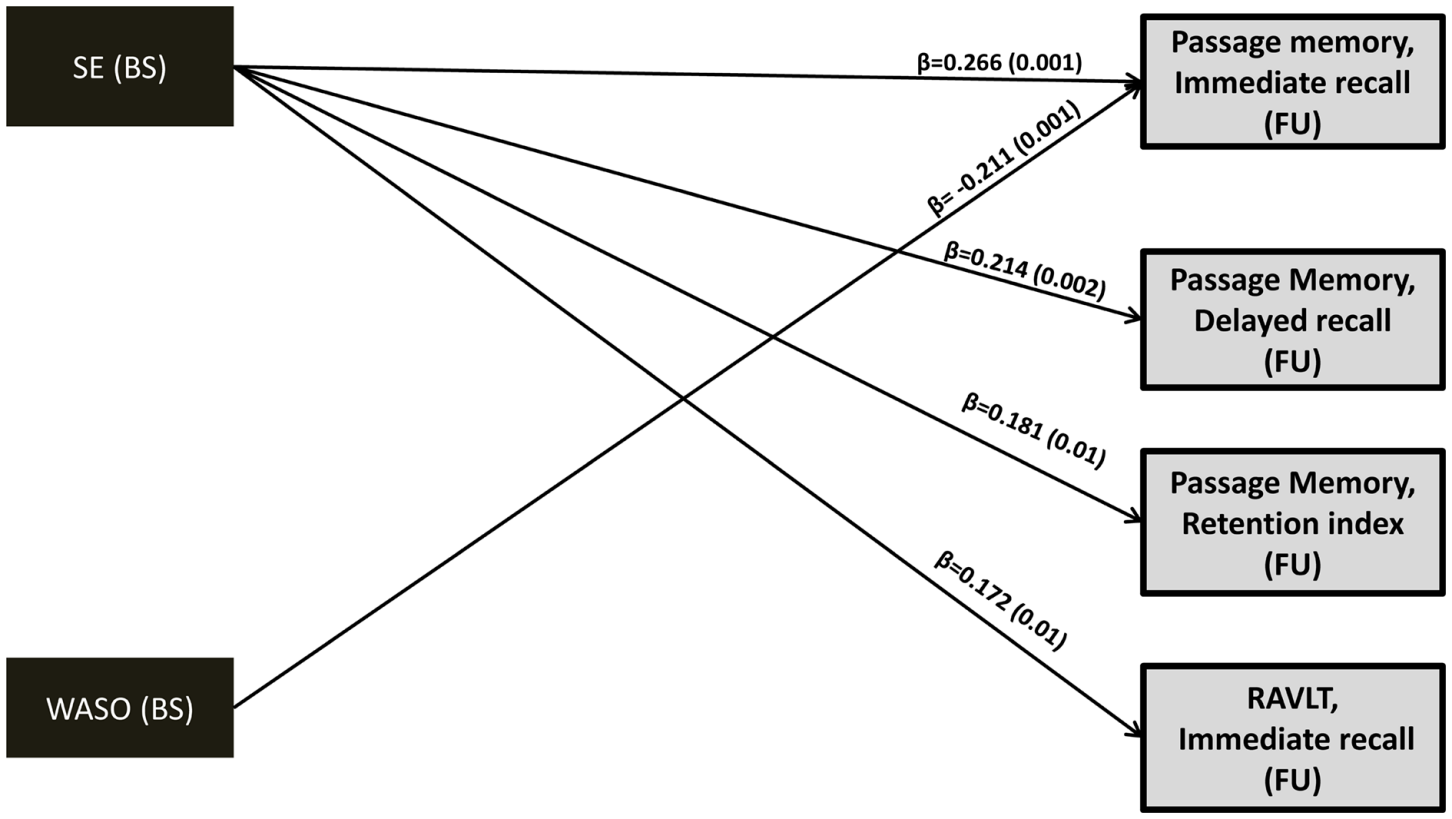
Direct effects (standardized regression coefficients; β) and *p* values (in parentheses) of baseline SE and WASO on follow-up memory performance (z-scores). Model fit indices as well as direct/indirect effects of baseline memory performance (z-scores) on subsequent memory performance can be found in Supplementary Table S1. Covariates included in the model (not shown): age, gender, education years, depression or anxiety diagnosis, insomnia-type symptoms, sleep apnea, number of medical morbidities, benzodiazepine use and BMI. BS, Baseline; FU, Follow-up; WASO, Wake After Sleep Onset time; SE, Sleep Efficiency; RAVLT, Rey Auditory Verbal Learning Test.

In models meeting all fit criteria we found significant *direct effects* of baseline SE and WASO on both immediate and delayed passage recall (*p* ≤ 0.01; see Figure 2 and Supplementary Table S1). In addition, baseline memory performance was significantly associated with subsequent memory performance in all models (Supplementary Table S1). Higher baseline SE was associated with better follow-up memory performance after controlling for age, gender, education years, depression/anxiety diagnosis, BMI, insomnia symptoms, medical comorbidities, self-reported symptoms of sleep apnea and benzodiazepine use (as recorded at baseline). The clinical significance of this association is highlighted by inspection of corresponding unstandardized regression coefficients. Thus, an increase by 1 percentage point in SE would predict an increase of 0.031, 0.027 and 0.051 SD in immediate and delayed recall as well as memory retention capacity, respectively. Similarly, an increase in baseline WASO duration by 1 min would predict a decline by 0.005 SD in immediate recall capacity. Significant direct effects of baseline sleep quality indices were found in one additional model demonstrating marginally acceptable fit (SE on RAVLT immediate recall: b = 0.026, β = 0.172, *p* = 0.01; see also Supplementary Table S1).

#### 3.5.2. Prolonged 24-h sleep duration and TiB over time predicts verbal episodic memory decline

**Figure 3:**
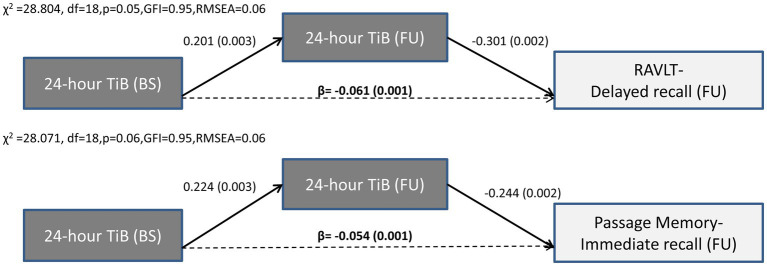
Results of path models illustrating the mediated effect of baseline 24-h TiB on follow-up word list (RAVLT) and Passage memory performance through 24-h TiB. Direct (solid arrows) and indirect effects (dotted arrows) are expressed as standardized regression coefficients (p values in parentheses). All models take into account the covariance between baseline sleep and memory variables, the direct and indirect paths from baseline memory to follow-up memory scores (see also Supplementary Table S1), and the following covariates (not shown): age, gender, education years, depression or anxiety diagnosis, insomnia symptoms, BMI, sleep apnea, benzodiazepine use and medical morbidities. Model fit indices are also shown. BS, Baseline; FU, Follow-up; df, degrees of freedom; GFI, Goodness of fit index; RMSEA, Root Mean Square Error of Approximation; TiB, Total Time in Bed; RAVLT, Rey Auditory Verbal Learning Test.

**Figure 4:**
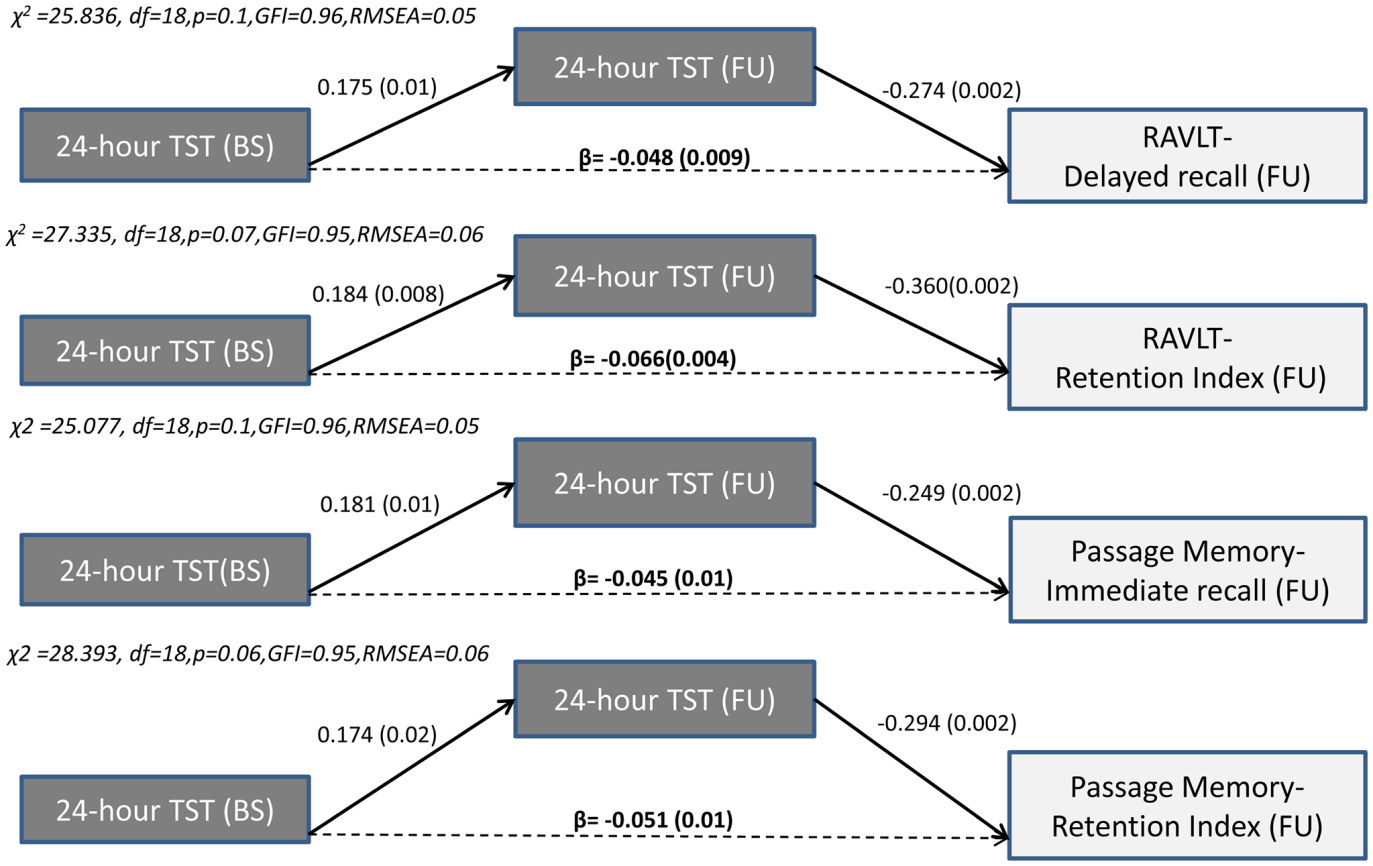
Results of path models illustrating the mediated effect of baseline 24-h TST on follow-up word list (RAVLT) and Passage memory performance through 24-h TST. Direct (solid arrows) and indirect effects (dotted arrows) are expressed as standardized regression coefficients (p values in parentheses). All models take into account the covariance between baseline sleep and memory variables, the direct and indirect paths from baseline memory to follow-up memory scores (see also Supplementary Table S1), and the following covariates (not shown): age, gender, education years, depression or anxiety diagnosis, insomnia symptoms, BMI, sleep apnea, benzodiazepine use and medical morbidities. Model fit indices are also shown.BS, Baseline; FU, Follow-up; df, degrees of freedom; GFI, Goodness of fit index; RMSEA, Root Mean Square Error of Approximation; TST, Total Sleep Time; RAVLT, Rey Auditory Verbal Learning Test.

In contrast to the long-term effects of sleep quality indices, only *indirect effects* of baseline 24-h TiB and TST on subsequent verbal episodic memory were found. Thus, the negative association between TiB/TST and subsequent memory capacity was fully mediated by the long-term progression of these sleep indices, in the presence of significant associations between baseline memory performance and subsequent memory performance. Indirect effects persisted after controlling for demographics, baseline depression and anxiety diagnosis, insomnia symptoms and sleep apnea, medical morbidities and BMI, as well as benzodiazepine use. As illustrated in Figures 3, 4, longer baseline TiB and TST were associated with longer follow-up TiB and TST, respectively (as indicated by corresponding direct paths), which in turn, predicted lower scores on delayed word list recall (RAVLT; β = −0.061, *p* = 0.001 and β = −0.048, *p* = 0.009, respectively) and immediate passage memory recall (β = −0.054, p = 0.001 and β = −0.045, *p* = 0.01, respectively). As indicated by standardized regression coefficients of significant indirect effects (β; Figure 4), a combined one SD increase in baseline and follow-up TST is accompanied by a 0.066 and 0.051 SD reduction in RAVLT and Passage Memory retention scores, respectively.

Inspection of Supplementary Table S1 reveals several additional significant indirect effects of baseline TiB and TST on follow-up memory indices in the context of path models displaying marginally acceptable model fit (TST on immediate word list recall: β = −0.064, p = 0.01; TST on delayed Passage recall: β = −0.033, *p* = 0.01; TiB on delayed Passage recall: β = −0.044, p = 0.001; TiB on Passage retention: β = −0.071, *p* = 0.001 and TiB on word list retention index: β = −0.079, *p* = 0.003).

#### 3.5.3. Direct and indirect paths between baseline sleep indices and follow-up memory performance in each clinical group

**Figure 5:**
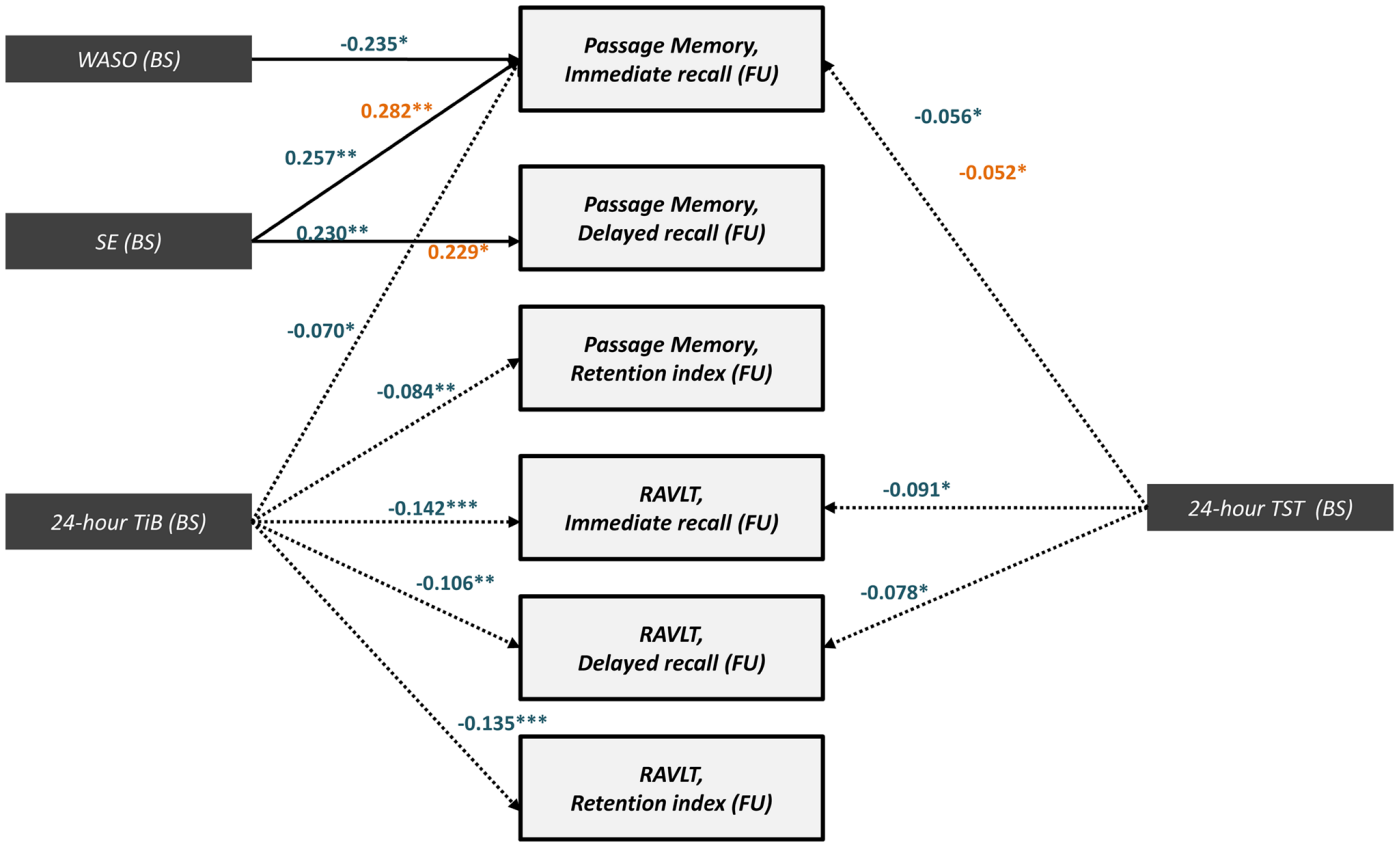
Significant direct (solid arrows) and indirect effects (dotted arrows) for MCI (blue letters) and CNI group (orange letters) expressed as standardized regression coefficients between baseline sleep indices and follow-up memory performance (z-scores). All models take into account the covariance between baseline sleep and memory variables, the direct and indirect paths from baseline memory to follow-up memory scores (see also Supplementary Table S2), and the following covariates (not shown): age, gender, education years, depression or anxiety diagnosis, insomnia symptoms, BMI, sleep apnea benzodiazepine use and medical morbidities. Model fit indices are presented in Supplementary Table S2. (***), *p* ≤ 0.001, (**), *p* ≤ 0.01, (*), *p* ≤ 0.05. BS, Baseline; FU, Follow-up; WASO, Wake After Sleep Onset time; SE, Sleep Efficiency; TiB, Total Time in Bed; TST, Total Sleep Time; RAVLT; Rey Auditory Verbal Learning Test.

Several of the effects described in sections 3.5.1–2 reached significance in models conducted separately in each clinical group (baseline diagnosis; Figure 5). Regarding sleep quality indices, there were significant (positive) *direct* effects of baseline SE on subsequent immediate (MCI: β = 0.257, SE = 0.097, p = 0.01; CNI:β = 0.282, SE = 0.120, *p* = 0.01) and delayed passage recall (MCI:β = 0.230, SE = 0.087, p = 0.01 and CNI:β = 0.229, SE = 0.121, *p* = 0.04, respectively). In the MCI group there was an additional (negative) direct effect of WASO on immediate passage recall (β = −0.235, SE = 0.099, *p* = 0.02).

Conversely, the full mediation of the effects of baseline sleep quantity indices (TST and TiB) on immediate/delayed recall and retention scores by follow-up TST and TiB was predominantly found among MCI persons. In this group, increased baseline TST predicted lower follow-up memory scores through follow-up TST (Passage immediate recall: β = −0.056, SE = 0.042, *p* = 0.03; RAVLT- immediate recall: β = −0.091, SE = 0.058, *p* = 0.03; RAVLT- delayed recall: β = −0.078, SE = 0.050, *p* = 0.02). The indirect effects of baseline TiB on memory performance also reached significance (Figure 5). In the CNI group, significant indirect effects were restricted to baseline TST on follow-up immediate passage recall (β = −0.052, SE = 0.032, p = 0.04). In all models, effects remained significant in the presence of significant direct effects of baseline memory on follow-up memory scores as well as after controlling for demographics, insomnia symptoms, depression or anxiety diagnosis, sleep apnea, medical morbidities and use of benzodiazepines and BMI. It should be noted, however, that these models displayed slightly lower (still acceptable) GFI values compared to those observed in the total sample (see Supplementary Table S2), assumingly due to the smaller sample sizes in the group-stratified analyses.

The aforementioned results were supported by two sets of supplementary univariate analyses. Firstly, CNI “progressors” spent more time in bed at both time points and had increased sleep duration that reached significance only at follow-up, compared to “non-progressors” (see Supplementary Table S3). MCI “progressors” spent more time in bed and displayed longer 24-h sleep duration (only at follow-up), and also showed lower SE and higher WASO at baseline.

Secondly, longer 24-h TiB and WASO at baseline was associated with greater reduction of memory scores in the total sample and separately in each group (larger baseline minus follow-up memory scores indicate greater decline in memory capacity). Also, lower baseline sleep efficiency was associated with a greater decline in memory performance, especially among MCI persons, as indicated by significant, modest correlations (r > −0.4; see Supplementary Table S4 and Supplementary Figures S1–S3).

## 4. Discussion

The main finding of the present study is that prolonged sleep and/or poor sleep quality in elderly predicts poor, long-term episodic verbal memory outcomes. The pathways through which these effects were noted varied according to the type of sleep parameter. Specifically, *lower SE and higher WASO* at baseline directly predicted poor memory outcomes over a period of 7–9 years. Conversely, the effects of baseline *sleep quantity* and *time in bed* were fully mediated by corresponding sleep indices at follow-up. As expected, age- and education-adjusted memory performance declined significantly over time, affecting all memory indices (immediate recall, delayed recall and retention index), and was more pronounced among persons initially diagnosed with MCI.

### 4.1. Higher baseline sleep efficiency and WASO directly predict future verbal memory performance

The direct effects of night sleep efficiency on the 7-9-year verbal memory outcomes were found in the total sample as well as among MCI and CNI persons and involved several memory measures assessing encoding, retrieval and consolidation processes. In addition, higher baseline WASO was associated with increased difficulty in encoding new structured verbal material (short passages). Importantly, these effects were present after controlling for initial memory performance, as well as for the concurrent associations between memory and sleep quality indices at both measurement points. The path analysis models employed in the present study included a number of potentially crucial demographic and clinical covariates (age, education, gender, BMI, depression/anxiety diagnosis, physical multicomorbidities and use of benzodiazepines, insomnia-type symptoms and sleep apnea at baseline). When interpreting these results, one should consider that objective sleep quality indices generally improved over time in both clinical groups, probably as a result of increased antidepressant use following baseline assessment within the MCI (25.3% vs. 43.6% at follow-up, *p* = 0.006) and CNI groups (34.8% vs. 42.0% at follow-up, *p* < 0.001). Furthermore, the observed increase in the SE index may stem from an increase in 24-h TST on follow-up (by 35 min on average), especially among MCI persons, as a considerable number progressed to dementia on Phase III (44.3%). However, in the absence of indirect effects of sleep quality indices on later memory capacity, we cannot conclude that improved sleep quality had a protective role on memory decline over time. Instead it is more plausible that relatively poor baseline sleep quality indices characterized persistent disrupted sleep at the time of initial measurement. Such sleep patterns may have already impacted neurobiological mechanisms supporting memory function at that time, increasing vulnerability for further memory decline over the following years.

To date, the majority of actigraphy studies have mainly emphasized the negative association of sleep discontinuity on executive function scores. Increased objective sleep quality measures predicted improved outcomes in conceptual flexibility tasks (Bernstein et al., 2019) and measures of global cognition (Djonlagic et al., 2021). Lower performance on working memory tasks was associated with fragmented sleep indices (sleep efficiency status <85% and increased WASO>30 min; Miyata et al., 2013; Cavuoto et al., 2016). A recent cross-sectional study (Cabanel et al., 2019) conducted in a small group of patients with MCI or dementia yielded significant associations between poor sleep efficiency (<85%) and impaired memory/language performance, whereas results of a longitudinal study conducted in a representative sample of community-dwellers without cognitive impairment (mean age = 70.5 years at baseline) highlight the contribution of both WASO and efficiency on future cognitive decline (McSorley et al., 2019). The current findings establish the potential deleterious effects of diminished sleep quality on all stages of episodic memory processing and suggest that adequate SE in elderly may help preserve future memory function.

In contrast to our findings, a cross-sectional study (André et al., 2019) failed to detect significant associations between objectively assessed sleep fragmentation and cognitive performance among MCI persons aged 63–80 years, whereas the effect of sleep discontinuity on cognitive impairment (executive deficits) was more prominent among healthy individuals of similar age. Also, a recent meta-analysis of both actigraphy and polysomnography studies, concluded that sleep continuity indices exhibited marginally significant, weak correlations with episodic memory performance, although there was evidence for a stronger moderating effect of WASO duration on the relationship between age and episodic memory performance in older adults as compared to younger individuals (Hokett et al., 2021). Although baseline WASO duration in our sample was higher compared to other actigraphy studies (Lysen et al., 2020; Buratti et al., 2021), significant effects of WASO were found only for immediate verbal recall among MCI persons (with additional marginally significant effects on delayed recall in both clinical groups). Methodological issues such as different sample size and study design, demographic characteristics (fewer years of formal schooling of the MCI group), neuropsychological tests and indices used (free recall vs. recognition measures; associative vs. non-associative memory tasks), and the shorted actigraphic period used in the present study may have led to the aforementioned discrepancies.

Accumulating evidence highlights the bidirectional associations between diminished sleep quality and the pathophysiology of AD (Chylinski et al., 2022). Concerning the underlying mechanism involved in the longitudinal detrimental effects of sleep quality on memory capacity, it has been proposed that increased sleep fragmentation contributes to the incremental accumulation—from middle age—of pathophysiological biomarkers (Aβ deposition and tau protein) involved in late life cognitive impairment and dementia-associated pathology (Scullin and Bliwise, 2015; Molano et al., 2017). Sleep macrostructure indices, such as awakenings occurring during sleep stage transitions, have been implicated in Aβ burden from the early phase of AD pathology, assumingly leading to disrupted proteinosynthesis and/or disorganization of oscillation coupling which could in turn disrupt memory consolidation (Chylinski et al., 2021, 2022). Lastly, increased WASO may appear to moderate the relationship between Aβ deposition and deficits in immediate/delayed recall, independently of neurocognitive status (Wilckens et al., 2018).

### 4.2. Increased 24-h sleep duration over time predicts verbal memory decline

In contrast to sleep quality indices, the negative impact of longer 24-h TST and TiB at baseline was fully mediated by each of the two sleep quantity indices, as measured over a period of 7–9 years. Although these effects were found in the total sample, they were more pronounced, and involved a wider range of memory scores, assessing encoding, retrieval and consolidation processes, in the MCI group. Interestingly, longitudinal trends of sleep quantity indices were opposite to those observed for sleep quality: TiB and TST significantly increased in the MCI group, whereas stability (TST) and even reduction (TiB) was noted in the CNI group. The finding of full mediation in our path models indicates that memory capacity at follow-up was mainly determined by the (positive) relation of sleep quantity indices between the two time points (adjusting for baseline memory performance). Although causality may not be concluded on the basis of observational, albeit longitudinal data, taken together these findings are consistent with the notion that memory decline over time was accounted for by concurrent increases in TiB and/or TST.

Although these indirect effects were found with both sleep indices, they were more extensive (i.e., affecting a wider range of memory scores) for TST, highlighting the role of prolonged sleep instead of mere physical inactivity. Importantly, these effects were significant after controlling for correlations between memory, TST and TiB at baseline (in addition to a set of potentially crucial demographic and clinical covariates, including insomnia-type symptoms as baseline).

It remains unclear whether objectively/subjectively assessed long or short sleep duration constitute risk factors for cognitive impairment, as some studies report high risk for cognitive deterioration and MCI/dementia incidence for both short (<6 h) and long (>8 h) sleepers (based on self-reports), suggesting a U-shaped relationship (Chen et al., 2016). In our sample, average baseline and follow-up sleep duration reached 7.3 and 7.9 h, respectively. Despite the fact that sleep duration within the CNI group remained stable, on average, between the two measurement points and within the normal range (7.5 h), persons who displayed higher TST by 1 SD on either baseline and/or follow up ended up with reduced short-term verbal memory z-scores (by 0.052 SDs). It should be noted that two previous, large cross-sectional studies failed to establish significant associations between TST and memory performance among cognitively intact individuals (Basta et al., 2019; Scarlett et al., 2021). In line with our findings, longitudinal studies examining subjectively assessed sleep patterns and cognitive progression reported significant, although relatively weak associations between increasing 24-h TST and verbal memory decline in community-dwelling elderly (Gildner et al., 2019; Zhang et al., 2023).

Among persons initially diagnosed with MCI, 24-h TST and TiB increased, on average, by 67 and 30 min, reaching 8.2 and 9.5 h, respectively, on a daily basis. In agreement with the present study, Kimura et al. (2019), reported increasing odds of global cognitive decline for an average sleep duration that exceeds 7 h. Moreover, long sleep duration (>8 h) was associated with executive and working memory deficits, as well as verbal memory decline in older adults (Cavuoto et al., 2016; Okuda et al., 2021). Self-reported increased sleep duration was linked to memory deficits among cognitively intact and MCI Greek community-dwellers (Tsapanou et al., 2017). The long-term, accumulated effect of sleep quantity (as measured at both measurement points) on memory performance indicates that sleep quantity may impact cognitive progression in the long run but also represents a marker of ongoing disease severity.

Sleep and cognition are closely interrelated processes. According to the “amyloid cascade hypothesis” dysregulation of the sleep–wake cycle may contribute to Aβ (and even tau-protein) accumulation either through prolonged periods of wakefulness and consequent increase in neuronal activity (Cedernaes et al., 2017) or *via* disorganization of clearance processes (Zhang et al., 2021). Cognitive impairment and increased levels of cerebrospinal fluid Aβ levels usually accompany sleep dysregulation in cross-sectional studies (Cedernaes et al., 2017). Sleep duration may contribute to cortical thinning by exerting differential effects on frontal (prolonged sleep, >7 h), and temporal areas (<7 h; Spira et al., 2016). Intraindividual variability in sleep patterns may affect both brain regions leading to disorganization in brain network function, which is crucial for memory consolidation. Furthermore, effects on dorsolateral and ventrolateral prefrontal areas that are actively engaged in encoding, efficient memory tracing and retrieval of information could further exacerbate memory deficits (Van der Linden et al., 2000). At a more global level, more stable sleep-activity patterns may contribute to reduced Aβ burden and, in turn, to better cognitive outcomes even in patients who already display mild cognitive deficits (Fenton et al., 2023).

### 4.3. Clinical implications

The findings of the current study highlight the significant impact that particular sleep characteristics may exert on long-term memory outcomes. Memory decline is probably one of the earliest and most easily noticeable signs in neurocognitive disorders, pervading daily life and affecting functionality. In the present study, both objectively assessed poor sleep quality and increased sleep duration were associated with memory decline, whereas sleep quantity indices were significant correlates of memory capacity at each time point, among persons demonstrating objective cognitive impairment as well as among cognitively intact elderly. Therefore, interventions should focus on improvement of both quality and quantity sleep indices at the pre-clinical phase, but also throughout the disease progression while, simultaneously, taking into account critical factors that may synergistically undermine sleep quality (multicomorbidity, emotional/anxiety disorders, chronic pain, sleep-related breathing disorders). Up to now, behavioral and combined sleep-based interventions are often implemented in elderly at risk for cognitive deterioration or persons diagnosed with neurocognitive disorders (Cordone et al., 2021). Such interventions may include: timely management of psychiatric symptomatology (depression and anxiety) through customized pharmacological treatment regiments (i.e., non-sedative vs. sedative), psychotherapy targeting sleep (sleep hygiene protocols in the context of CBT-insomnia; CBT-i) combined with behavioral changes (social activities and physical exercise programs) and systematic monitoring of medical conditions associated with sleep discontinuity (e.g., obstructive sleep apnea). Such sleep-based interventions were found efficacious in improving sleep efficiency and WASO among “normal” elderly (Martin et al., 2017) and provided promising findings concerning executive function improvement among MCI patients (Cassidy-Eagle et al., 2018). Also, the concurrent use of trazodone and implementation of sleep strategies (CBT-i) may prove particularly effective in regulating cortisol levels and lengthen sleep duration, in cases of inadequate sleep duration (Vgontzas et al., 2020).

### 4.4. Strengths and limitations

Although the interplay between sleep and memory function is well established and extensively studied at the molecular and cellular levels, evidence on specific associations between sleep–wake patterns and memory processes in normal aging and neurocognitive disorders (MCI; dementia) is sparse and based on subjective estimates of sleep disturbances and measures of global cognition instead of specialized neuropsychological tests. Our study encompasses objective actigraphy measures at two time points, a comprehensive neuropsychological examination, neuropsychiatric assessment, and medical/functionality information, affording a well-characterized profile of cognitive progression, clinical status characterization, and description of sleep–wake patterns. Also, the longitudinal design with a relatively long follow-up period allows for a systematic monitoring of variables of interest among community-dwellers, including a rather large sample of MCI participants. The presence of a control group (cognitively intact individuals) matching the study group in terms of certain demographic factors, permits a direct comparison with individuals diagnosed with MCI and allows for distinct sleep and cognition trajectories to unravel in the long term.

The current study is not without limitations. Stratifying participants by initial neurocognitive status reduced sample size and may have led to underestimation of associations between sleep and cognition. Importantly, although the unusually long follow-up period may have increased (unmeasured) variability in potentially crucial variables (such as comorbidities), and significantly impacted attrition rate due to incapacitating illness or death, it afforded the unique opportunity to assess very long-term effects of sleep on cognition. In addition, commonly utilized goodness of fit estimates is strictly dependent on sample size and as a result, violation of goodness of fit averted us from reporting models with suboptimal fitting parameters. Also, actigraphy is not the optimal method to differentiate between actual sleep and sedentary time. Furthermore, actigraphy was conducted for 3 24-h periods, while other studies recommend 1–2 week recordings. Finally, the presence of other sleep disorders such as sleep apnea known to be frequent in this age group and to be related with cognitive function (Bixler et al., 1998), was based on self-report rather than the gold-standard method (polysomnography).

### 4.5. Conclusion

In conclusion, our study demonstrated that objective long sleep duration and poor sleep efficiency predict memory performance in elderly without dementia. We suggest that in this population, early interventions focusing on sleep may help improve memory capacity and delay memory decline.

## Data availbility statement

The raw data supporting the conclusions of this article will be made available by the authors, without undue reservation.

## Ethics statement

The studies involving humans were approved by Heraklion University Hospital Research Ethics Committee. The studies were conducted in accordance with the local legislation and institutional requirements. Written informed consent for participation in this study was provided by the participants' legal guardians/next of kin.

## Author contributions

ES: Conceptualization, Data curation, Formal analysis, Investigation, Methodology, Visualization, Writing – original draft, Writing – review & editing. PS: Conceptualization, Formal analysis, Methodology, Supervision, Writing – review & editing. AZ: Data curation, Investigation, Writing – review & editing. EK: Data curation, Investigation, Writing – review & editing. IZ: Methodology, Supervision, Writing – review & editing. CA: Conceptualization, Data curation, Methodology, Writing-review & editing. AV: Conceptualization, Funding acquisition, Methodology, Supervision, Writing – review & editing. MB: Conceptualization, Funding acquisition, Methodology, Supervision, Writing – review & editing.
