# Supplementary material for: Long-term associations between objective sleep quality and quantity and verbal memory performance in normal cognition and mild cognitive impairment
Source: Front Neurosci. 2023 Oct 19;17:1265016. doi: 10.3389/fnins.2023.1265016 (PMC10620682; doi:10.3389/fnins.2023.1265016)
Supplement: Supplementary file 1 [file Data_sheet_1.docx]

Supplementary Table 1. Model fit indices for the 16 models where significant direct and/or indirect effects of baseline sleep on follow-up memory scores were found in the present study (total sample).

| **Outcome**  **(at follow up)** | **Primary predictor**  **(at baseline)** | **x^2^ (df), p value** | **GFI** | **RMSEA** | **Direct effect (baseline sleep on follow-up memory ;β, p value)** | **Direct effect (follow-up sleep on follow-up memory;β, p value)** | **Indirect effect (baseline sleep on follow-up memory;β, p value)** | **Direct effect (baseline memory on follow-up memory ;β, p value)** | **Indirect effect (baseline memory on follow-up memory; β, p value)** |
| --- | --- | --- | --- | --- | --- | --- | --- | --- | --- |
| ***AVLT-Immediate recall*** | **SE** | 34.558(18), 0.01 | 0.95 | 0.08 | 0.172 (0.01) | -0.043(0.5) | -0.012(0.4) | 0.698 (0.001) | - |
|  | **24-hour TST** | 37.647(18), 0.004 | 0.94 | 0.08 | -0.014(0.8) | -0.372(0.001) | -0.064(0.01) | 0.404 (0.001) | - |
| ***AVLT-Delayed recall*** | **24-hour-TiB** | 28.804(18),0.05 | 0.95 | 0.06 | -0.070 (0.3) | -0.301(0.002) | -0.061 (0.001) | 0.518 (0.002) | 0.062(0.001) |
|  | **24-hour TST** | 25.836(18), 0.1 | 0.96 | 0.05 | -0.033(0.6) | -0.274(0.002) | -0.048(0.009) | 0.540 (0.002) | 0.049 (0.01) |
| ***AVLT-Retention Index*** | **24-hour TiB** | 29.919(18), 0.03 | 0.95 | 0.06 | -0.028(0.7) | -0.410(0.002) | -0.079(0.003) | 0.325 (0.001) | 0.100 (0.001) |
|  | **24-hour TST** | 27.335(18), 0.07 | 0.95 | 0.06 | -0.015(0.9) | -0.360(0.002) | -0.066(0.004) | 0.368 (0.001) | 0.072 (0.005) |
| ***PM- Immediate recall*** | **SE** | 23.869(18),0.1 | 0.96 | 0.04 | 0.266(0.001) | -0.122(0.1) | -0.035(0.05) | 0.519 (0.002) | - |
|  | **WASO** | 25.965(18), 0.1 | 0.96 | 0.05 | -0.211(0.001) | -0.046(0.4) | -0.009(0.3) | 0.520 (0.002) | 0.004 (0.3) |
|  | **24-hour TiB** | 28.071(18), 0.06 | 0.95 | 0.06 | -0.080(0.2) | -0.244(0.002) | -0.054(0.001) | 0.497 (0.002) | 0.034 (0.01) |
|  | **24-hour TST** | 25.077(18),0.1 | 0.96 | 0.05 | 0.040(0.5) | -0.249(0.002) | -0.045(0.01) | 0.496 (0.001) | 0.029 (0.06) |
| ***PM- Delayed recall*** | **SE** | 27.449(18), 0.07 | 0.96 | 0.06 | 0.214(0.002) | -0.028(0.6) | -0.008(0.5) | 0.638 (0.002) | -0.000 (0.8) |
|  | **24-hour TiB** | 32.638(18), 0.01 | 0.95 | 0.07 | -0.092(0.1) | -0.212(0.002) | -0.044(0.001) | 0.620 (0.002) | 0.025 (0.07) |
|  | **24-hour TST** | 29.419(18), 0.04 | 0.95 | 0.06 | 0.0008(0.9) | -0.192(0.003) | -0.033(0.01) | 0.635 (0.002) | 0.018 (0.1) |
| ***PM- Retention Index*** | **SE** | 26.010(18), 0.09 | 0.96 | 0.05 | 0.181(0.01) | 0.017(0.7) | 0.005(0.7) | 0.396 (0.002) | 0.001 (0.5) |
|  | **24-hour TiB** | 31.927(18), 0.02 | 0.95 | 0.07 | -0.020(0.7) | -0.343(0.002) | -0.071(0.001) | 0.393 (0.002) | 0.029 (0.4) |
|  | **24-hour TST** | 28.393(18), 0.06 | 0.95 | 0.06 | 0.047(0.5) | -0.294(0.002) | -0.051(0.01) | 0.412 (0.002) | 0.017 (0.4) |

Supplementary Table 2. Model fit indices for the models where significant direct/indirect effects of baseline sleep on follow-up memory scores were found, stratified by initial cognitive status (MCI vs CNI group).

| **Outcome**  **(at follow up)** | **Primary predictor**  **(at baseline)** | **x^2^ (df), p value** | **GFI** | **RMSEA** | **Direct effect (baseline sleep on follow-up memory;β, p value)** | **Direct effect (follow-up sleep on follow-up memory; β, p value)** | **Indirect effect (baseline sleep on follow-up memory; β, p value)** | **Direct effect (baseline memory on follow-up memory;β, p value)** | **Indirect effect (baseline memory on follow-up memory; β, p value)** |
| --- | --- | --- | --- | --- | --- | --- | --- | --- | --- |
| ***AVLT-Immediate recall*** | **24-hour TiB (MCI)** | 22.792(18),0.2 | 0.93 | 0.06 | -0.105(0.4) | -0.419(0.003) | -0.142(0.001) | 0.307 (0.001) | 0.049 (0.1) |
|  | **24-hour TST (MCI)** | 22.228(18),0.2 | 0.93 | 0.05 | -0.073(0.4) | -0.390(0.004) | -0.091(0.03) | 0.282 (0.009) | 0.035 (0.3) |
| ***AVLT-Delayed recall*** | **24-hour TiB (MCI)** | 18.925(18),0.4 | 0.94 | 0.02 | 0.023(0.9) | -0.331(0.003) | -0.106(0.002) | 0.308 (0.009) | 0.030 (0.2) |
|  | **24-hour TST (MCI)** | 19.303(18),0.4 | 0.94 | 0.03 | 0.048(0.8) | -0.329(0.001) | -0.078(0.02) | 0.314 (0.006) | 0.027 (0.4) |
| ***AVLT-Retention Index*** | **24-hour TiB (MCI)** | 24.991(18),0.1 | 0.92 | 0.07 | 0.022(0.9) | -0.446(0.0008) | -0.135(0.0008) | 0.100 (0.03) | 0.066 (0.08) |
| ***PM- Immediate recall*** | **SE (MCI)** | 17.028(18), 0.5 | 0.95 | 0.00 | 0.257(0.01) | -0.110(0.2) | -0.205(0.1) | 0.348 (0.002) | 0.005 (0.3) |
|  | **SE (CNI)** | 22.587(18),0.2 | 0.92 | 0.06 | 0.282(0.01) | -0.263(0.06) | -0.110(0.03) | 0.457 (0.002) | 0.007 (0.5) |
|  | **WASO (MCI)** | 19.728(18), 0.3 | 0.95 | 0.03 | -0.235(0.02) | 0.003(0.9) | 0.0006 (0.8) | 0.325 (0.002) | -0.000 (0.8) |
|  | **24-hour TiB (MCI)** | 22.800(18),0.2 | 0.93 | 0.06 | -0.006(0.8) | -0.221(0.03) | -0.070(0.02) | 0.321 (0.003) | -0.003 (0.7) |
|  | **24-hour TST(MCI)** | 23.429(18),0.2 | 0.93 | 0.06 | 0.163(0.2) | -0.238(0.03) | -0.056(0.03) | 0.322 (0.003) | -0.009 (0.5) |
|  | **24-hour TST (CNI)** | 24.484(18),0.1 | 0.92 | 0.07 | -0.107(0.3) | -0.224(0.05) | -0.052(0.04) | 0.463 (0.002) | -0.002 (0.8) |
| ***PM- Delayed recall*** | **SE (MCI)** | 13.589(18), 0.7 | 0.96 | 0.00 | 0.230(0.01) | -0.018(0.7) | -0.003(0.5) | 0.555 (0.000) | 0.000 (0.7) |
|  | **SE (CNI)** | 26.748(18),0.08 | 0.92 | 0.08 | 0.229(0.04) | -0.111(0.3) | -0.046(0.2) | 0.522 (0.001) | 0.000 (0.9) |
| ***PM- Retention Index*** | **24-hourTiB (MCI)** | 19.282(18),0.4 | 0.94 | 0.03 | 0.030(0.9) | -0.268(0.007) | -0.084(0.005) | 0.322 (0.000) | 0.012 (0.4) |

Supplementary Table 3. Baseline sleep parameters (mean [SD]) for subgroups of MCI and CNI participants: those who displayed significant cognitive deterioration during the study period (“progressors”) and those who maintained their cognitive status (“non-progressors”).

|  | **CNI progressors (n=33)** | | **CNI non-progressors (n=36)** | | **MCI non-progressors (n=44)** | | **MCI progressors (n=35)** | |
| --- | --- | --- | --- | --- | --- | --- | --- | --- |
|  | **Baseline** | **Follow-up** | **Baseline** | **Follow-up** | **Baseline** | **Follow-up** | **Baseline** | **Follow-up** |
| **Sleep efficiency** | 82.2(7.9) | 86.8(4.5) | 83.7(5.75) | 86.0(4.6) | 81.5(8.5)^4^ | 84.9(5.6) | 77.3(9.11)^4^ | 85.5(6.41) |
| **WASO** | 79.4(37.3) | 62.2(21.9) | 72.2(32.5) | 56.6(19.2) | 71.2(35.6)^5^ | 67.5(27.04) | 92.4(44.67)^5^ | 78.3(35.21) |
| **24-hour TiB** | 581.7(59.6)^1^ | 557.30(91.7)^2^ | 538.8(91.3)^1^ | 488.7(50.7)^2^ | 534.1(88.8) | 535.3(65.8)^3^ | 566.1(86.07) | 635.8(75.42)^3^ |
| **24-hour TST** | 468.0 (54.3) | 483.7(83.7)^6^ | 438.1(73.4) | 424.4(48.4)^6^ | 427.0(62.0) | 458.5(63.4)^7^ | 433.6(66.42) | 543.9(80.0)^7^ |

Note: Pairs of means where significant differences were found (p<0.05) are marked with the same superscript.

Supplementary Table 4. Correlations between decline in memory scores (Baseline minus follow-up raw score) and sleep parameters measured at baseline or follow-up.

|  | **Sleep efficiency** | | **WASO** | | **24-hour TiB** | | **24-hour TST** | |
| --- | --- | --- | --- | --- | --- | --- | --- | --- |
|  | **Baseline** | **Follow-up** | **Baseline** | **Follow-up** | **Baseline** | **Follow-up** | **Baseline** | **Follow-up** |
| **Total sample** |  |  |  |  |  |  |  |  |
| RAVLT: immediate recall | **-0.215 (0.03)** | -0.064 | 0.201 | 0.185 | **0.233 (0.02)** | **0.371 (<0.001)** | 0.076 | **0.322 (0.002)** |
| RAVLT: delayed recall | -0.093 | 0.014 | 0.132 | 0.027 | 0.134 | 0.204 | 0.064 | 0.181 |
| RAVLT: retention capacity | -0.023 | -0.025 | 0.018 | 0.079 | 0.058 | **0.242 (0.03)** | 0.038 | 0.201 |
| Passage Memory: immediate recall | **-0.317 (0.002)** | 0.026 | **0.317 (0.002)** | 0.057 | **0.213 (0.03)** | **0.215 (0.04)** | 0.017 | 0.203 |
| Passage Memory: delayed recall | **-0.320 (0.002)** | -0.095 | **0.271 (0.008)** | 0.175 | 0.180 | **0.245 (0.02)** | -0.009 | 0.188 |
| Passage Memory: retention capacity | -0.121 | -0.147 | 0.133 | **0.248 (0.02)** | 0.102 | **0.308 (0.004)** | 0.027 | 0.225 |
| **MCI** |  |  |  |  |  |  |  |  |
| RAVLT: immediate recall | -0.264 | -0.024 | 0.264 | 0.170 | **0.380 (0.008)** | **0.508 (0.001)** | 0.205 | **0.435 (0.004)** |
| RAVLT: delayed recall | -0.040 | 0.029 | -0.002 | -0.004 | 0.091 | 0.096 | 0.043 | 0.082 |
| RAVLT: retention capacity | 0.016 | 0.147 | -0.057 | -0.069 | 0.089 | 0.195 | 0.118 | 0.231 |
| Passage Memory: immediate recall | **-0.447 (0.001)** | 0.012 | **0.340 (0.01)** | 0.056 | 0.123 | 0.250 | -0.200 | 0.214 |
| Passage Memory: delayed recall | **-0.457 (0.001)** | -0.203 | **0.341 (0.02)** | 0.229 | 0.091 | 0.137 | -0.186 | 0.037 |
| Passage Memory: retention capacity | -0.092 | -0.111 | 0.032 | 0.145 | -0.001 | 0.080 | -0.076 | 0.013 |
| **CNI** |  |  |  |  |  |  |  |  |
| RAVLT: immediate recall | -0.103 | -0.004 | 0.100 | 0.040 | 0.115 | 0.075 | 0.037 | 0.078 |
| RAVLT: delayed recall | -0.148 | 0.057 | **0.329 (0.02)** | -0.022 | 0.234 | **0.281 (0.05)** | 0.151 | 0.265 |
| RAVLT: retention capacity | 0.011 | -0.070 | 0.076 | 0.074 | 0.131 | 0.184 | 0.123 | 0.131 |
| Passage Memory: immediate recall | -0.191 | 0.046 | **0.300 (0.04)** | 0.077 | **0.306 (0.03)** | 0.228 | 0.212 | 0.224 |
| Passage Memory: delayed recall | -0.203 | 0.055 | 0.220 | 0.053 | **0.286 (0.05)** | 0.265 | 0.157 | 0.261 |
| Passage Memory: retention capacity | -0.165 | 0.002 | **0.325 (0.02)** | 0.086 | **0.363 (0.01)** | **0.294 (0.04)** | **0.282 (0.05)** | 0.278 |

Note: p values of significant correlations (p<0.05) in parentheses.


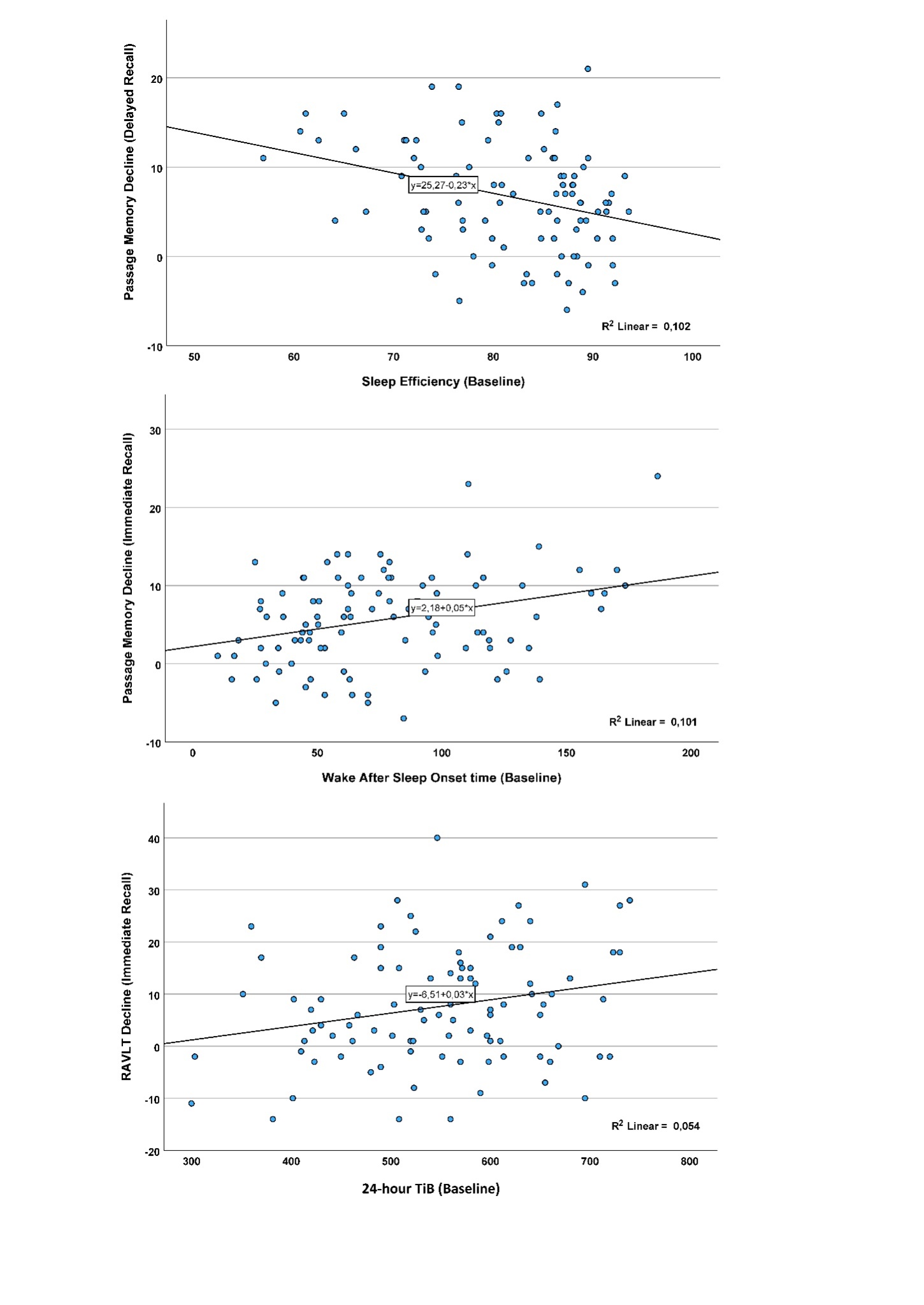
Supplementary Figure 1. Schematic illustration of the significant association between memory decline (Baseline minus follow-up raw scores) and sleep parameters measured at baseline, in the total sample. Upper panel: Sleep efficiency, middle panel: wake after sleep onset time, lower panel: 24-hour time in bed (TiB).


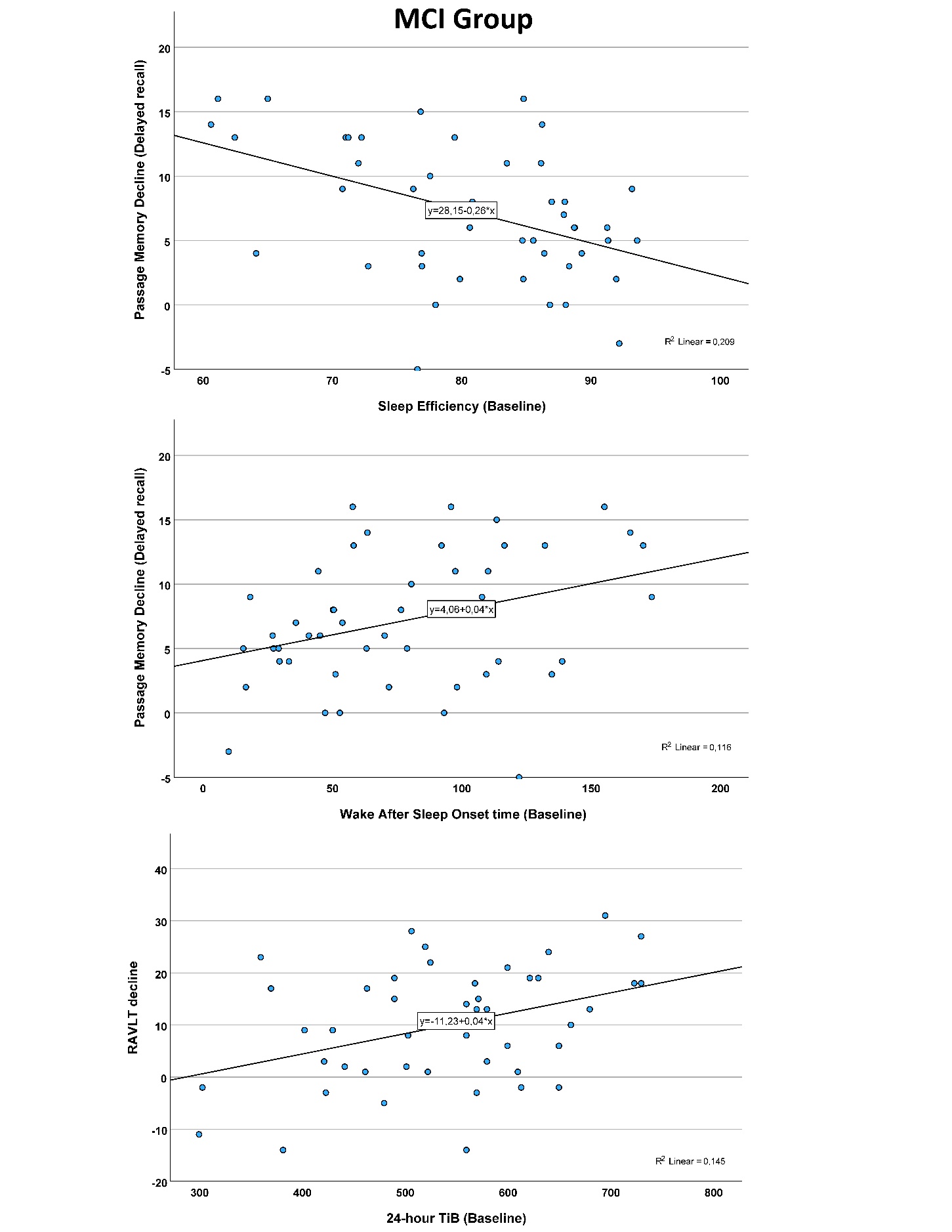


Supplementary Figure 2. Schematic illustration of the significant association between memory decline (Baseline minus follow-up raw scores) and sleep parameters measured at baseline, among participants diagnosed as MCI at baseline. Upper panel: Sleep efficiency, middle panel: wake after sleep onset time, lower panel: 24-hour time in bed (TiB).


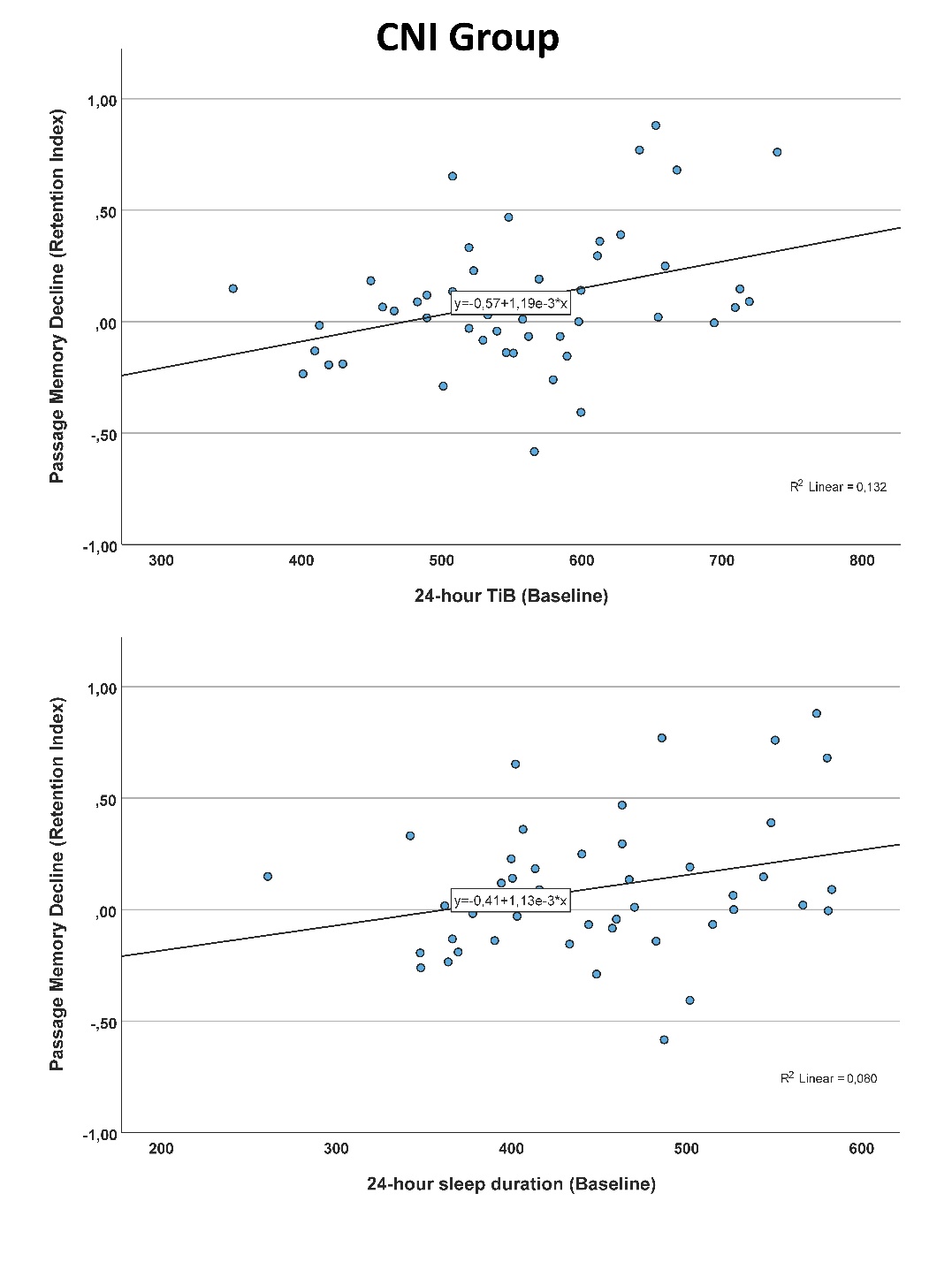


Supplementary Figure 3. Schematic illustration of the significant association between memory decline (Baseline minus follow-up raw scores) and sleep parameters measured at baseline, among participants found to be cognitively non-impaired (CNI) at baseline. Upper panel: 24-hour time in bed (TiB), lower panel: 24-hour total sleep time.
